# CBCT Evaluation of Periapical Pathologies in Maxillary Posterior Teeth and Their Relationship with Maxillary Sinus Mucosal Thickening

**DOI:** 10.3390/healthcare11060787

**Published:** 2023-03-07

**Authors:** Rizwan Jouhar, Hussain Mohammed Alkhames, Muhammad Adeel Ahmed, Naji Mohammad Almadeh, Muhammad Faheemuddin, Muhammad Farooq Umer

**Affiliations:** 1 Department of Restorative Dental Sciences, College of Dentistry, King Faisal University, Al-Ahsa 31982, Saudi Arabia; 218008282@student.kfu.edu.sa (H.M.A.); mshakeel@kfu.edu.sa (M.A.A.); 217019692@student.kfu.edu.sa (N.M.A.); 2 Department of Operative Dentistry and Endodontics, Altamash Institute of Dental Medicine, Karachi 75500, Pakistan; 3 Department of Prosthodontics and Implantology, College of Dentistry, King Faisal University, Al-Ahsa 31982, Saudi Arabia; mnaseem@kfu.edu.sa; 4 Department of Preventive Dental Sciences, College of Dentistry, King Faisal University, Al-Ahsa 31982, Saudi Arabia; mumer@kfu.edu.sa

**Keywords:** oral health, cone-beam computed tomography, maxillary permanent first molar, mucosal thickening of maxillary sinus, periapical pathology

## Abstract

In modern dentistry, radiographic imaging is crucial for examining the connection between the maxillary sinus floor and the root apices of the maxillary posterior teeth, particularly when the periapical region is affected by pathology that could result in infectious, inflammatory, or traumatic changes in the maxillary sinus. This study aimed to investigate the prevalence of periapical pathologies in the maxillary posterior teeth and their relationship with maxillary sinus mucosal thickening by using cone-beam computed tomography scans. This retrospective study was conducted on 420 digitized CBCT images which were scanned in sagittal, axial, and coronal views. Out of 420 total images, 223 (53.1%) were of males and 197 (44.9%) were of females. The data were analyzed using SPSS version 28. A total of 2936 posterior maxillary teeth were tested for periapical pathology (PP), 1477 on the right side and 1459 on the left side. In terms of gender, there was no significant relationship between PP in maxillary posterior teeth on both sides and mucosal thickness of the maxillary sinus (*p* > 0.05). A significant relationship was found between PP in maxillary posterior teeth on both sides and maxillary sinus mucosal thickening (*p* < 0.05). This study concluded that the prevalence of periapical pathology in the maxillary posterior teeth was significantly associated with a rise in the incidence of maxillary sinus mucosal thickening. Moreover, the primary causative factor for the pathophysiology of the odontogenic maxillary sinus was the periapical pathology in both maxillary first molars.

## 1. Introduction

The periapical tissues undergo pathological changes as a result of infection from pulp necrosis spreading into the periapical area. The activation of host immune responses may result from interactions between the stimulants that leaked out of the canal space and the host response. The infection at the periapical region of the maxillary posterior teeth can spread to the maxillary sinus via the blood and lymphatic vessels and result in sinus diseases [1]. Depending on the severity of the tooth infection, these diseases can range from a simple sinusitis to severe rhino sinusitis and even brain abscess [2].

In the body of the maxillary bone, there is a pyramid-shaped air-filled cavity known as the maxillary sinus. The maxillary sinus is more prone to pathogenic germs than other paranasal sinuses because it is confined to the nasal cavity and mouth cavity. When the swelling of the membrane that covers the paranasal sinuses lasts for at least 12 weeks, it is referred to as chronic maxillary sinusitis. Oral pathogenic bacteria or the nasal ostium may be the source of chronic maxillary sinusitis [3].

For 10–12% of cases of maxillary sinusitis, odontogenic factors are responsible [4]. Odontogenic pathologies such as apical inflammatory lesions, endodontic problems, radicular cysts, and marginal periodontitis can cause odontogenic sinusitis [5]. The extrusion of root canal filling materials from the apex of associated teeth into the sinus cavity, which facilitated the transport of microorganisms from the periapical tissues to the maxillary sinuses, has been implicated in previous studies as the cause of odontogenic sinusitis caused by root canal treatment of posterior molar teeth [6,7].

Clinically, symptoms of odontogenic and nonodontogenic sinusitis are similar. However, because odontogenic sinusitis has a different microbiology than nonodontogenic sinusitis, the therapeutic approach for maxillary sinusitis should be based on where the infection initiated [8]. No research has considered the roles of several dental and maxillary sinus factors in the etiology of odontogenic sinusitis despite the fact that some prior studies focused on the etiology of odontogenic maxillary sinusitis [9,10], dental restorations, a tooth’s periapical and periodontal health, and the separation between the maxillary sinus mucosa and the base of the sinus mucosa. Recent research has shown associations between dental pathology and radiographic sinusitis signs, particularly mucosal thickening of Schneider membrane [1,7,11,12].

A variety of inflammatory symptoms can cause increased sinus mucosal thickness (SMT), and the influencing factors related to patients such as age or history of smoking; odontogenic factors for instance; occurrence of periapical lesions, the severity of periodontal infections, and the amount of lost alveolar bone; and morphological factors of maxillary sinus such as the measurement section and the sinus septa [9,13,14]. According to one study, 59.3% of patients with SMT of more than 5 mm had ostium blockage [15]. As the mucosa could only be seen at a thickness of 2 mm or higher, Cagici et al., Janner et al., and Shanbhag et al., reported that the anatomy of the paranasal cavity affected cases where the SMT > 2 mm; as a result, 2 mm was generally believed to be a reliable threshold for pathological mucosal swelling [16,17,18].

For a proper diagnosis and patient management to be made, it is crucial to understand the connection between odontogenic and sinusal diseases [19]. Odontogenic maxillary sinus is said to be underdiagnosed and frequently ignored in the otolaryngology literature, resulting in patients’ prolonged symptoms and the failure of medication and surgical sinusitis therapy [20,21].

To evaluate teeth and odontogenic infections, two-dimensional panoramic and intraoral X-rays can be used [22]. However, periapical and sinus abnormalities cannot be detected using two-dimensional imaging modalities. Panoramic radiographs can recognize fluid deposition or mucosal thickness (MT), but they cannot accurately detect the structure of the sinuses [20]. A high-precision method is essential to identify the sinus anatomy in its entirety. As compared to multi-slice computed tomographic imaging, cone-beam computed tomography (CBCT) imaging may provide high-quality three-dimensional images with a negligible radiation dosage [23,24]. It allows for a thorough evaluation of the maxillary bone quality and quantity without superimposing or misrepresenting the teeth and adjacent structures [23,24]. This is a great approach to evaluate the relations between the maxillary sinus and neighboring teeth, making it an excellent evaluation tool for patients with both dental and sinus problems [25].

Regarding CBCT images, the prevalence of odontogenic maxillary sinus diseases has been evaluated in a few studies [1,7,26,27]. However, these studies investigated only the thickness of the maxillary sinus mucosa rather than the relationships between the pathologies of the maxillary sinus and the periapical pathologies of the maxillary posterior teeth. Therefore, the purpose of this study was to determine the prevalence of periapical pathologies in the maxillary posterior teeth and their relationship with maxillary sinus diseases by using cone-beam computed tomography scans in the Al Ahsa region at the Dental Complex of King Faisal University. 

## 2. Materials and Methods

This retrospective study was piloted at Dental Complex of King Faisal University after obtaining ethical approval from the Research Ethics Committee of Deanship of Scientific Research at King Faisal University (KFU-REC-2022-OCT-ETHICS271). Data of patients who gave consent were retrieved from the record. A total of 420 digitized CBCT images from 2018 to 2021 were selected. Maxillary CBCT scans of patients between the ages of 16 and 60 that showed all first and second premolars as well as first and second molars that had fully erupted and established roots met the inclusion criteria. However, CBCT scans that revealed an orthodontic retainer or implant, bone anomalies, suspected tumors in the posterior region of the maxilla or in the maxillary sinus, a history of paranasal sinus diseases, respiratory disorders such as bronchitis or bronchial asthma, and incomplete demographic details were excluded from the study.

Demographic data were documented including patient age and gender. CBCT images were taken with I-CAT Vision QTM (Imaging Sciences International, Hat-field, PA, USA. Version 1.9.3.14) 360-degree rotation with a 130-degree field of view, 0.25 mm voxel size at 0.250–0.400 Res, 120 kV, and 5 mA with exposure times of 2–7 s. The gathering, interpretation, and recording of CBCT images were carried out by BlueSkyPlan (Version 4.7.55, GmbH, Langenhagen, Germany) software that had been used to identify the presence of mucosal thickening in the right and left sinuses and the presence of a periapical lesion in the posterior maxillary teeth. Initially, panoramic radiographs were used to consider general evaluation and the presence of posterior maxillary teeth. Following that, using the MPR view, specifically the coronal view, both the right and left sinuses as well as the periapical area were evaluated with over confirmation using the sagittal view. 

The first and second premolars, as well as the first, second, and third molars, on both the right and left sides of the maxilla were examined for peripheral pathology by two examiners with inter-examiner reliability (Cohen’s kappa) of 0.81. The presence or absence of opacity in the maxillary sinuses was evaluated in both the right and left maxillary sinuses.

The data were analyzed using SPSS version 28 (IBM Corp., Armonk, NY, USA). Descriptive statistics (frequencies and percentages) were used to express presence of periapical lesions and mucosal thickening of the maxillary sinus. An independent sample T test was used to compare the means of mucosal thickening of maxillary sinuses between males and females. Additionally, binary logistic regression was used for the prediction of maxillary sinus mucosal thickening due to presence/absence of periapical radiolucency (*p*-value 0.05 was considered statistically significant).

## 3. Results

A total of 420 CBCT images were evaluated, wherein 223(53.1%) were males and 197(44.9%) were females, with 2936 posterior maxillary teeth tested for PP, 1477 on the right side and 1459 on the left side. The presence of PP on the right side of the first premolar was observed in 85(20.2%) patients, of whom 55(64.7%) were males and 30(35.3%) were females, with a mean age of 40.8 ± 12 years. Furthermore, 103(24.5) patients had PP in their right second premolar; their mean age was 41.05 ±13.0 years, with a 55(55.3%) male predilection, as shown in Table 1. 

The status of PP of the left first premolar revealed that 66(15.7%) patients had PP with a mean age of 40.19 ± 12.3 years and a male predilection of 42(63.6%). Furthermore, 119(28.3%) patients had PP in the left second premolar, with a mean age of 39.40 ± 12.2 years and a male dominance of 71(59.7%), as shown in Table 2. 

Out of these 420 CBCT images, 840 maxillary sinuses (MS) were assessed for the presence of mucosal thickening in the maxillary sinus on both sides. On the right side, mucosal thickening was found in 126(56.5%) male patients and 89(45.2%) female patients, and no mucosal thickening was observed in the remaining 205(48.8%) patients, with an insignificant difference with respect to gender, (*p* = 0.499). Additionally, on the left side, mucosal thickening was found predominantly in 133(59.6%) male patients and 83(42.1%) female patients; no mucosal thickening was observed in the remaining 204(48.6%) patients, with an insignificant difference with respect to gender, (*p* = 0.467), as shown in Table 3.

The relationship between the maxillary sinus mucosal thickness and the periapical pathologies of the maxillary posterior teeth on the right side revealed that most of the patients (167(80.70%)) had PP and showed mucosal thickening, while 40(19.30%) patients had no PP but showed mucosal thickening. Furthermore, most of the patients (167(77.50%)) had no PP along with no mucosal thickening of maxillary sinus, while 48(22.50%) patients had PP but showed no mucosal thickening, With a significant difference among them, (*p* < 0.001). On the left side, most of the patients (171(79.2%)) who had PP showed mucosal thickening as well, while 46(22.5%) patients had no PP but showed mucosal thickening. Furthermore, most of the patients 158(77.50%) had no PP along with no mucosal thickening of maxillary sinus, while 45(20.8%) had PP but showed no mucosal thickening, with a significant difference among them, (*p* < 0.001), as shown in Table 4. 

**Figure 1:**
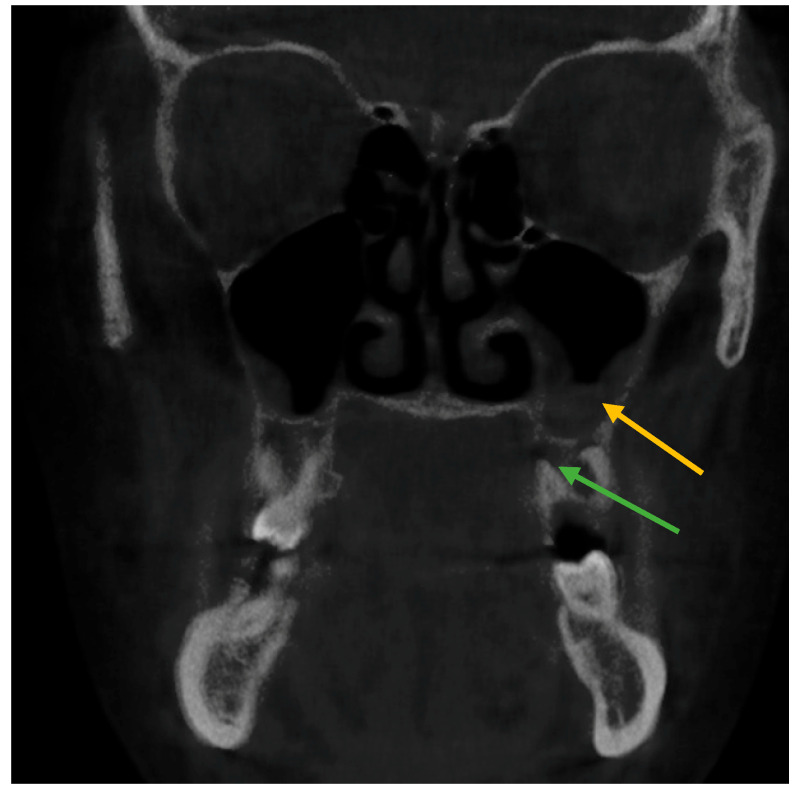
Changes in mucosal thickness in maxillary sinus observed in coronal view on CBCT imaging. (Yellow arrow shows mucosal thickening, green arrows show periapical infection).

**Figure 2:**
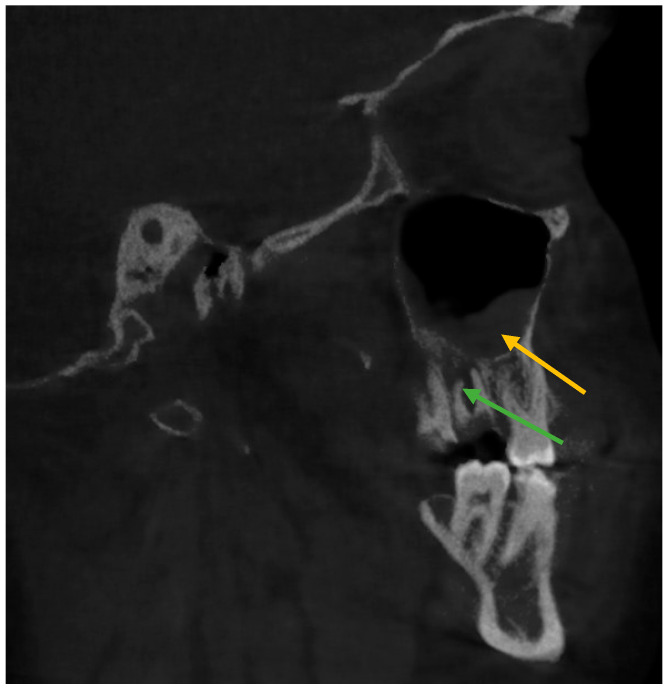
Changes in mucosal thickness in maxillary sinus observed in sagittal view on CBCT imaging. (Yellow arrow shows mucosal thickening, green arrows show periapical infection).

The relationship of mucosal thickening associated with periapical infection is shown in the coronal and sagittal view of CBCT images in Figure 1 and Figure 2, respectively.

## 4. Discussion

Odontogenic sinusitis must be comprehensively evaluated clinically and radiographs must be taken if desired for the diagnosis. Panoramic radiographs and particular skull views, such as Water’s projection, are some of the radiological types employed to envisage the maxillary sinus [28,29]. Owing to the non-specific nature of radiographic symptoms and the propensity of these modalities to hide the odontogenic origin, two-dimensional imaging has a restricted role in the diagnosis of sinusitis [30]. Innovative improvements in three-dimensional imaging technologies have opened up an extensive range of potential advantages for dentistry since they enable multiple section evaluation. The capability to collect thin slices, deliver multiplanar images for interactive viewing, and permit visualization of the bone and soft tissue make CT effective for maxillary sinus imaging [28]. The relation between a periapical lesion and the sinus floor may be demonstrated using axial and coronal sinus CT images [30]. The application of this technology in dentistry has been constrained by its comparatively expensive cost, high dosage of radiation, and limited availability to hospitals and medical radiology departments. The interest in using CT for an expanding variety of dental treatments has expanded considerably with the advent of comparatively low-cost and low-dose CBCT machines specifically designed for maxillofacial imaging [31,32]. Therefore, the present study demonstrated a relationship between periapical pathologies in the maxillary posterior teeth and the thickening of the mucosa in the maxillary sinus using CBCT images.

Numerous studies have established the value of CBCT imaging in demonstrating the etiology and degree of the connection between a dental disease and sinus involvement [7,26]. The present study corroborated the aforementioned advantages of CBCT imaging in accurately identifying the odontogenic origin of the mucosal thickness of the maxillary sinus.

The present study reported an insignificant relationship between age, gender, and maxillary sinus pathology. However, it was also shown by the present study that mostly maxillary sinus pathology was present in the younger age group with male predilection, with an insignificant relationship (*p* > 0.001). These findings were consistent with other studies, which found no connection between age and the incidence of maxillary sinus disease [33,34]. 

Remarkably, the present study showed that PP was present among the younger age group with male dominancy as compared to females, but an insignificant association existed. These findings of the present research were partially corroborated with the research by Phothikhun et al. [35] and Vallo et al. [11], who revealed a greater incidence of mucosal thickening among older age groups and males than younger age groups and females, respectively. Additionally, another study found that males were more likely than females to have maxillary sinus disease, with an insignificant association with age [36]. Males had a considerably higher prevalence of maxillary sinus disease than females. Similar to this, the majority of earlier investigations revealed that males had a considerably increased prevalence of the maxillary sinus disease compared to females [11,26,35].

The roots of the maxillary posterior dentition are located so near to the sinus floor that pulpal inflammation or infection might compromise the health of the sinus floor. When teeth with root apices that are near or extend into the maxillary sinuses develop periapical lesions, this may cause inflammatory changes in the mucosal lining, which may lead to sinusitis [1,7]. Additionally, it is thought that bacteria and toxins in apical lesions might infect the sinus mucosa of the maxillary sinuses either directly or through the several vascular anastomoses, spongy alveolar bone marrow, and lymphatic system. The severity of apical lesions might therefore grow due to such increases in the quantity of bacteria and toxins, which can also lead to an increase in the maxillary sinus pathology [1,7]. The results of the present study showed that the majority of patients with PP of maxillary posterior teeth on the right side 167(80.70%) and on the left side 171(79.2%) were more likely to develop mucosal thickening in the maxillary sinus, which indicated maxillary sinusitis with odontogenic origin. The present results did not line up with earlier research, which found that the frequency of odontogenic maxillary sinusitis in the general population ranged from 10–12% [3,37]. The results of the present study, however, were supported by further recent studies using CT or CBCT that showed maxillary sinusitis with an odontogenic origin is relatively common and that up to 86% of sinusitis patients may have an odontogenic cause [1,7,12,26,29].

A 51.8% incidence of odontogenic maxillary sinusitis was also demonstrated by Maillet et al. [1], which was incompatible with the results of the present study that showed about 80.70% of patients on the right side and 79.2% of patients on the left side had PP that caused mucosal thickening of maxillary sinus. The present results were similar to the research by Obayashi et al., who discovered that maxillary sinus changes were connected to tooth infection in 71.3% of patients [29]. Similarly, the prevalence of right and left odontogenic maxillary sinusitis was reported to be 59.5% and 64%, respectively, in a different study by Kasikcioglu A., which was not consistent with the present study. Males were more likely to have maxillary sinus disease, and age had no relationship with maxillary sinus disease [36]. 

Apical periodontitis and other odontogenic diseases have also been linked to maxillary sinusitis. As a periapical lesion becomes more severe, the incidence of maxillary sinus mucosal thickening substantially rises [38]. The results of the present study indicated that mucosal thickening of the maxillary sinus was significantly more common when PP was present in the maxillary molar teeth on both sides (*p* < 0.001). These findings support another study that found a substantial increase in the prevalence of maxillary sinus disease on both the left and right sides in the presence of PP in the maxillary posterior teeth [36].

The maxillary sinus typically connects to the roots of maxillary teeth from the second premolar to the third molar based on anatomical site [39]. The present study reported that the prevalence of PP was most frequently observed in maxillary first molars on both sides as its root is closer to the floor of the maxillary sinus and causes mucosal thickening. These conclusions were supported by the study of Maillet et al., who found that the maxillary first molar was the tooth most frequently linked to the thickening of the maxillary sinus mucosa [1]. The maxillary second molar’s mesiobuccal root, however, was closest to the sinus, according to the results of a CT scan [28].

Interestingly, in other research, CBCT images were used to assess the impact of apical lesions on the degree of mucosal thickening and apical bone thickness [40]. Only a limited number of studies have examined whether mucosal thickening exists in relation to apical lesions [7,41]. Their results disagree with those of an earlier study, which found that the presence of teeth with apical lesions increased the amount of mucosal thickening [41]. This can be explained by the fact that a number of variables that contribute to infection spread also have an impact on mucosal thickening [42]. Furthermore, the number of teeth in this area and the anatomic proximity—that is, the distance between the maxillary sinus and the apex of the roots of posterior teeth—also actively contribute to the thickening of the mucosa. Additionally, the thinning of the mucosa may be indirectly aided by the aging-related increase in tooth loss, alveolar crest atrophy, and sinus pneumatization [43]. The results of the present study were in line with the findings of the abovementioned studies and showed that the presence of periapical diseases facilitates the spread of infection that results in the thickening of the mucosa in the maxillary sinus. However, the non-odontogenic cause of maxillary sinus disease was not evaluated in the present study.

In addition, evidence from a few earlier investigations shows that mucosal thickness increases with bone resorption [9,44]. Non-odontogenic causes have also reportedly been shown to promote mucosal thickening. Odontogenic infections in the palatal roots of molars can readily migrate through the lateral wall of the maxillary sinus due to the porous nature of the maxillary bone, which may result in mucosal thickening [45]. The reason for this is because molar roots are anatomically closer to the maxillary sinus than premolar roots. These findings were endorsed by another study [40]. The present study showed consistency and reported that periapical pathology caused by maxillary first molars increased the prevalence of mucosal thickening of the maxillary sinus as the roots of first molars are in close proximity to the maxillary sinus.

Similarly, the present study found that the roots of the molars are anatomically closer to the maxillary sinus than the roots of the premolars; thus, PP in the first molars on both sides dramatically increased the chances of infection spreading to the maxillary sinus, resulting in increased maxillary sinus mucosal thickness. These findings were consistent with subsequent research. In assessing the relationship between the tooth type and mucosal thickening, the extent of mucosal thickening in the molars was observed to be greater as compared to the premolars [1,46]. Further research exploring the relationship between the type of tooth and the presence of lesions reveals that lesions are frequently identified in the first and second molar teeth [7,40,46,47].

Consequently, to increase the likelihood of a successful therapeutic outcome, CBCT is an efficient, high-precision three-dimensional imaging technique that accurately identifies the mucosal thickening of the maxillary sinus brought on by the odontogenic infection.

This study had certain limitations. Firstly, the availability of complete information on various sociodemographic variables such as history of smoking and time of infection was lacking. Our study banked on retrospective data, so this gap can be addressed in the subsequent in-depth study. In addition, Khojastepour et al. [48] found an extension of the maxillary sinus in 68.8% and 2.5% of canine teeth and incisors, respectively, among the Iranian population; these extended sinus images were not included in our study and can be an area of interest in future studies on the Saudi population. Other drawbacks included that we did not investigate the likelihood of non-odontogenic maxillary sinusitis coinciding with periapical disease of a maxillary posterior tooth; the frequency of odontogenic maxillary sinusitis may have been overstated in our study; and due to the retrospective re-search design, we were unable to assess whether the maxillary sinus disease had healed or not. In order to determine the incidence of odontogenic maxillary sinus dis-ease detectable on CBCT scans and connect these findings to the clinical pathologies of maxillary posterior teeth, prospective studies are required.

## 5. Conclusions

This study concluded that the prevalence of periapical pathology in the maxillary posterior teeth was significantly associated with the rise in the incidence of maxillary sinus mucosal thickening. The primary causative factor for the pathophysiology of the odontogenic maxillary sinus was the periapical lesions in both maxillary first molars. Additionally, males were more likely to have periapical disease, but there was no significant difference with respect to gender in the mucosal thickening of the maxillary sinus. Therefore, future prospective research is needed to determine the frequency of odontogenic maxillary sinus disease that is apparent on cone-beam computed tomography scans and to link these results to clinical disorders of the maxillary posterior teeth.

## Figures and Tables

**Table 1:** Prevalence of periapical pathology of maxillary posterior teeth on the right side.

Right Side	Status of Tooth		Total *n* (%)	Age Mean ± SD	Gender	
					Male	Female
**First Premolar**	**Periapical Pathology**	**Present**	85(20.2)	40.8 ± 12	55(64.7%)	30(35.3%)
		**Absent**	291(69.1)	33.38 ± 12	150(51.5)	141(48.5)
		**Missing**	44(10.5)	44.45 ± 10	18 (40.9)	26(59.1)
**Second Premolar**	**Periapical Pathology**	**Present**	103(24.5)	41.05 ± 13	57(55.3)	46(44.7)
		**Absent**	258(61.3)	32.39 ± 11	131(50.8)	127(49.2)
		**Missing**	59(14)	43.24 ± 10	35(59.3)	24(40.7)
**First Molar**	**Periapical Pathology**	**Present**	145(34.4)	39.17 ± 12	75(51.7)	70(48.3)
		**Absent**	212(50.4)	32.17 ± 11	109(51.4)	103(48.6)
		**Missing**	63(15)	41.87 ± 11	39(61.9)	24(38.1)
**Second Molar**	**Periapical Pathology**	**Present**	99(23.5)	39.10 ± 13	49(49.5)	50(50.5)
		**Absent**	284(67.5)	33.61 ± 12	157(55.3)	127(44.7)
		**Missing**	37(8.8)	46.49 ± 10	17(45.9)	20(54.1)

**Table 2:** Prevalence of periapical pathology of maxillary posterior teeth on the left side.

Left Side	Status of Tooth		Total *n* (%)	Age Mean ± SD	Gender	
					Male	Female
**First Premolar**	**Periapical Pathology**	**Present**	66(15.7)	40.19 ± 12	42(63.6)	24(36.4)
		**Absent**	312(74.3)	33.92 ± 12	164(52.6)	148(47.4)
		**Missing**	42(10)	45.31 ± 10	17(40.5)	25(59.5)
**Second Premolar**	**Periapical Pathology**	**Present**	119(28.3)	39.40 ± 12	71(59.7)	48(40.3)
		**Absent**	244(58.1)	32.26 ± 11	124(50.8)	120(49.2)
		**Missing**	57(13.6)	45.21 ± 11	28(49.1)	29(50.9)
**First Molar**	**Periapical Pathology**	**Present**	128(30.5)	38.23 ± 12	66(51.5)	62(48.4)
		**Absent**	207(49.3)	31.67 ± 11	109(52.7)	98(47.3)
		**Missing**	85(20.2)	43.38 ± 12	48(56.5)	37(43.5)
**Second Molar**	**Periapical Pathology**	**Present**	113(26.9)	41.01 ± 12	57(50.4)	56(49.6)
		**Absent**	270(64.3)	32.62 ± 11	145(53.7)	125(46.3)
		**Missing**	37(8.8)	45.84 ± 10	21(56.8)	16(43.2)

**Table 3:** Association of periapical pathology of maxillary posterior teeth on both sides with mucosal thickening of maxillary sinus with respect to gender.

Mucosal Thickening of Maxillary Sinus							
		Present, *n* (%)	Absent, *n* (%)	Mean ± SD	T	df	*p* Value
**Right side**							
**Gender**	**Male**	126(56.5)	97(43.5)	1.43 ± 0.497	−2.326	i418	0.499
	**Female**	89(45.2)	108(54.8)	1.55 ± 0.499			
	**Total**	215(51.2)	205(48.8)				
**Left side**							
**Gender**	**Male**	133(59.6)	90(40.4)	1.40 ± 0.492	−3.630	418	0.467
	**Female**	83(42.1)	114(57.9)	1.58 ± 0.495			
	**Total**	216(51.4)	204(48.6)				

**Table 4:** Association of periapical pathology of maxillary posterior teeth with mucosal thickening of maxillary sinus.

			Mucosal Thickening of Maxillary Sinus			
			Present	Absent	*p*-Value	B
**Periapical Pathology**	**Right Side**	**Present**	167(80.70)	48(22.50)	<0.001	−2.664
		**Absent**	40(19.30)	165(77.50)		
	**Left Side**	**Present**	171(79.2)	45(20.8)	<0.001	−2.569
		**Absent**	46(22.5)	158(77.5)		

## Data Availability

The data presented in this study are available on request from the corresponding author. The data are not publicly available due to ethical concerns.

## References

[1] Maillet M., Bowles W.R., McClanahan S., John M.T., Ahmad M. (2011). Cone-beam computed tomography evaluation of maxillary sinusitis. J. Endod..

[2] Vijayan A., Sreejith V., Surendran R., Ahamed G. (2012). Orbital abscess arising from an odontogenic infection. J. Contemp. Dent. Pract..

[3] Mehra P., Murad H. (2004). Maxillary sinus disease of odontogenic origin. Otolaryngol. Clin. N. Am..

[4] Yildirim D., Eroglu M., Salihoglu M., Yildirim A.O., Karagoz H., Erkan M. (2013). The relationship between dental indentation and maxillary sinusitis. Open J. Med. Imaging.

[5] Little R.E., Long C.M., Loehrl T.A., Poetker D.M. (2018). Odontogenic sinusitis: A review of the current literature. Laryngoscope Investig. Otolaryngol..

[6] Kuan E.C., Suh J.D. (2017). Systemic and odontogenic etiologies in chronic rhinosinusitis. Otolaryngol. Clin. N. Am..

[7] Lu Y., Liu Z., Zhang L., Zhou X., Zheng Q., Duan X., Zheng G., Wang H., Huang D. (2012). Associations between maxillary sinus mucosal thickening and apical periodontitis using cone-beam computed tomography scanning: A retrospective study. J. Endod..

[8] Nash D., Wald E. (2001). Sinusitis. Pediatr. Rev..

[9] Goller-Bulut D., Sekerci A.E., Köse E., Sisman Y. (2015). Cone beam computed tomographic analysis of maxillary premolars and molars to detect the relationship between periapical and marginal bone loss and mucosal thickness of maxillary sinus. Med. Oral Patol. Oral Cir. Bucal.

[10] Tassoker M. (2020). What are the risk factors for maxillary sinus pathologies? A CBCT study. Oral Radiol..

[11] Vallo J., Suominen-Taipale L., Huumonen S., Soikkonen K., Norblad A. (2010). Prevalence of mucosal abnormalities of the maxillary sinus and their relationship to dental disease in panoramic radiography: Results from the Health 2000 Health Examination Survey. Oral Surg. Oral Med. Oral Pathol. Oral Radiol. Endod..

[12] Brüllmann D.D., Schmidtmann I., Hornstein S., Schulze R.K. (2012). Correlation of cone beam computed tomography (CBCT) findings in the maxillary sinus with dental diagnoses: A retrospective cross-sectional study. Clin. Oral Investig..

[13] Marin S., Kirnbauer B., Rugani P., Payer M., Jakse N. (2019). Potential risk factors for maxillary sinus membrane perforation and treatment outcome analysis. Clin. Implant Dent. Relat. Res..

[14] Cayo-Rojas C.F., Begazo-Jiménez L.A., Romero-Solórzano L.B., Nicho-Valladares M.K., Gaviria-Martínez A., Cervantes-Ganoza L.A. (2020). Periapical lesions and their relationship to Schneider’s membrane in cone-beam computed tomography. Int. J. Dent..

[15] Maska B., Lin G.H., Othman A., Behdin S., Travan S., Benavides E., Kapila Y. (2017). Dental implants and grafting success remain high despite large variations in maxillary sinus mucosal thickening. Int. J. Implant Dent..

[16] Cagici C.A., Yilmazer C., Hurcan C., Ozer C., Ozer F. (2009). Appropriate interslice gap for screening coronal paranasal sinus tomography for mucosal thickening. Eur. Arch. Otorhinolaryngol..

[17] Janner S.F.M., Caversaccio M.D., Dubach P., Sendi P., Buser D., Bornstein M.M. (2011). Characteristics and dimensions of the Schneiderian membrane: A radiographic analysis using cone beam computed tomography in patients referred for dental implant surgery in the posterior maxilla. Clin. Oral Implant. Res..

[18] Shanbhag S., Shanbhag V., Stavropoulos A. (2014). Volume changes of maxillary sinus augmentations over time: A systematic review. Int. J. Oral Maxillofac. Implant..

[19] Nascimento E.H., Pontual M.L.A., Pontual A.A., Freitas D.Q., Perez D.E.C., Ramos-Perez F.M. (2016). Association between odontogenic conditions and maxillary sinus disease: A study using cone-beam computed tomography. J. Endod..

[20] Patel N.A., Ferguson B.J. (2012). Odontogenic sinusitis: An ancient but under-appreciated cause of maxillary sinusitis. Curr. Opin. Otolaryngol. Head Neck Surg..

[21] Longhini A.B., Ferguson B.J. (2011). Clinical aspects of odontogenic maxillary sinusitis: A case series. Int. Forum Allergy Rhinol..

[22] Nunes C.A., Guedes O.A., Alencar A.H.G., Peters O.A., Estrela C.R., Estrela C. (2016). Evaluation of periapical lesions and their association with maxillary sinus abnormalities on cone-beam computed tomographic images. J. Endod..

[23] Liang X., Jacobs R., Hassan B., Li L., Pauwels R., Corpas L., Souza P.C., Martens W., Shahbazian M., Alonso A. (2010). A comparative evaluation of cone beam computed tomography (CBCT) and multi-slice CT (MSCT): Part I On subjective image quality. Eur. J. Radiol..

[24] Ezzodini Ardakani F., Razavi S.H., Tabrizizadeh M. (2015). Diagnostic value of cone-beam computed tomography and periapical radiography in detection of vertical root fracture. Iran. Endod. J..

[25] Cymerman J.J., Cymerman D.H., O’Dwyer R.S. (2011). Evaluation of odontogenic maxillary sinusitis using cone-beam computed tomography: Three case reports. J. Endod..

[26] Ritter L., Lutz J., Neugebauer J., Scheer M., Dreiseidler T., Zinser M.J., Rothamel D., Mischkowski R.A. (2011). Prevalence of pathologic findings in the maxillary sinus in cone-beam computerized tomography. Oral Surg. Oral Med. Oral Pathol. Oral Radiol. Endod..

[27] Bajoria A.A., Sarkar S., Sinha P. (2019). Evaluation of Odontogenic Maxillary Sinusitis with Cone Beam Computed Tomography: A Retrospective Study with Review of Literature. J. Int. Soc. Prev. Community Dent..

[28] Nair U.P., Nair M.K. (2010). Maxillary sinusitis of odontogenic origin: Cone-beam volumetric computerized tomography-aided diagnosis. Oral Surg. Oral Med. Oral Pathol. Oral Radiol. Endod..

[29] Obayashi N., Ariji Y., Goto M., Izumi M., Naitoh M., Kurita K., Shimozato K., Ariji E. (2004). Spread of odontogenic infection originating in the maxillary teeth: Computerized tomographic assessment. Oral Surg. Oral Med. Oral Pathol. Oral Radiol. Endod..

[30] Shahbazian M., Jacobs R. (2012). Diagnostic value of 2D and 3D imaging in odontogenic maxillary sinusitis: A review of literature. J. Oral Rehabil..

[31] Ludlow J.B., Timothy R., Walker C., Hunter R., Benavides E., Samuelson D.B., Scheske M.J. (2015). Effective dose of dental CBCT—A meta analysis of published data and additional data for nine CBCT units. Dentomaxillofac. Radiol..

[32] Ludlow J.B., Ivanovic M. (2008). Comparative dosimetry of dental CBCT devices and 64-slice CT for oral and maxillofacial radiology. Oral Surg. Oral Med. Oral Pathol. Oral Radiol. Endod..

[33] Drumond J.P.N., Allegro B.B., Novo N.F., Miranda S.L., Sendyk W.R. (2017). Evaluation of the Prevalence of Maxillary Sinuses Abnormalities through Spiral Computed Tomography (CT). Int. Arch. Otorhinolaryngol..

[34] Raghav M., Karjodkar F.R., Sontakke S., Sansare K. (2014). Prevalence of incidental maxillary sinus pathologies in dental patients on conebeam computed tomographic images. Contemp. Clin. Dent..

[35] Phothikhun S., Suphanantachat S., Chuenchompoonut V., Nisapakultorn K. (2012). Cone-beam computed tomographic evidence of the association between periodontal bone loss and mucosal thickening of the maxillary sinus. J. Periodontol..

[36] Kasikcioglu A., Gulsahi A. (2016). Relationship between maxillary sinus pathologies and maxillary posterior tooth periapical pathologies. Oral Radiol..

[37] Brook I. (2006). Sinusitis of odontogenic origin. Otolaryngol. Head Neck Surg..

[38] Kuligowski P., Jaroń A., Preuss O., Gabrysz-Trybek E., Bladowska J., Trybek G. (2021). Association between Odontogenic and Maxillary Sinus Conditions: A Retrospective Cone-Beam Computed Tomographic Study. J. Clin. Med..

[39] Fry R.R., Patidar D.C., Goyal S., Malhotra A. (2016). Proximity of maxillary posterior teeth roots to maxillary sinus and adjacent structures using Denta scan. Indian J. Dent..

[40] Kocak N., Alpoz E., Boyacioglu H. (2018). Evaluation of the effect of apical lesion on mucosal thickening and thickness of apical bone using limited cone-beam computed tomography. Niger. J. Clin. Pract..

[41] Bornstein M.M., Lauber R., Sendi P., von Arx T. (2011). Comparison of periapical radiography and limited cone-beam computed tomography in mandibular molars for analysis of anatomical landmarks before apical surgery. J. Endod..

[42] Yusufoglu S.I., Erbasar G.N.H., Gülen O. (2021). Evaluation of the effect of periapical lesions and other odontogenic conditions on maxillary sinus mucosal thickness characteristics and mucosal appearance: A CBCT study. J. Dent. Res. Dent. Clin. Dent. Prospect..

[43] Alqahtani S., Alsheraimi A., Alshareef A., Alsaban R., Alqahtani A., Almgran M., Eldesouky M., Al-Omar A. (2020). Maxillary Sinus Pneumatization following Extractions in Riyadh, Saudi Arabia: A Cross-sectional Study. Cureus.

[44] Cao Z., Yuan J. (2021). Changes in Maxillary Sinus Mucosal Thickening following the Extraction of Teeth with Advanced Periodontal Disease: A Retrospective Study Using Cone-Beam Computed Tomography. BioMed Res. Int..

[45] Psillas G., Papaioannou D., Petsali S., Dimas G.G., Constantinidis J. (2021). Odontogenic maxillary sinusitis: A comprehensive review. J. Dent. Sci..

[46] Shanbhag S., Karnik P., Shirke P., Shanbhag V. (2013). Association between periapical lesions and maxillary sinus mucosal thickening: A retrospective cone-beam computed tomographic study. J. Endod..

[47] Alhujhuj R.R., Jouhar R., Ahmed M.A., Almujhim A.A., Albutayh M.T., Adanir N. (2022). Evaluation of Root Canal Configuration of Maxillary and Mandibular First Molar by CBCT: A Retrospective Cross-Sectional Study. Diagnostics.

[48] Khojastepour L., Movahhedian N., Zolghadrpour M., Mahjoori-Ghasrodashti M. (2021). Assessment of the relationship between the maxillary sinus and the canine root tip using cone beam computed tomography. BMC Oral Health.

